# High-Speed Burring with and without the Use of Surgical Adjuvants in the Intralesional Management of Giant Cell Tumor of Bone: A Systematic Review and Meta-Analysis

**DOI:** 10.1155/2010/586090

**Published:** 2010-07-27

**Authors:** H. Algawahmed, Robert Turcotte, F. Farrokhyar, M. Ghert

**Affiliations:** ^1^Department of Surgery, McMaster University, Hamilton, ON, Canada L8V 5C2; ^2^Department of Orthopedic Surgery, McGill University Health Center, Montreal, Québec, Canada

## Abstract

Local control rates for Giant Cell Tumor of Bone (GCT) have been reported in a large number of retrospective series. However, there remains a lack of consensus with respect to the need for a surgical adjuvant when intralesional curettage is performed. We have systematically reviewed the literature and identified six studies in which two groups from the same patient cohort were treated with intralesional curettage and high-speed burring with or without a chemical or thermal adjuvant. Studies were evaluated for quality and pooled data was analyzed using the fixed effects model. Data from 387 patients did not indicate improved local control with the use of surgical adjuvants. Given the available data, we conclude that surgical adjuvants are not required when meticulous tumor removal is performed.

## 1. Introduction

Giant Cell Tumor of Bone (GCT) is a primary bone tumor of mesenchymal origin presenting as a localized osteolytic lesion. GCT typically affects the meta-epiphyseal region of long bones, mainly the distal femur and the proximal tibia with a peak incidence in the 3rd and 4th decades of life [1, 2]. Despite the fact that it is classified as a benign bone tumor, GCT exhibits locally aggressive features with an unpredictable course of progression [1, 3, 4].

Although close to 100% local control is achieved with en bloc resection [5], this type of procedure is commonly associated with functional disabilities due to the peri-articular location of many of GCTs [6]. Hence, intralesional curettage has been widely accepted as the standard of care for GCT of bone. This method of treatment carries a significantly higher recurrence rate with various recurrence rates ranging from approximately 13% to 49% [2, 5, 7–9]. In an attempt to reduce these high local recurrence rates, several toxic or thermal adjuvants have been advocated to provide local control. These include phenol, polymethylmethacrylate (PMMA), argon beam coagulation, anhydrous alcohol, and liquid nitrogen [10–15]. Although PMMA can be used as structural filler, it is believed to cause thermal injury to local cells and therefore acts as a surgical adjuvant [16]. 

There are, however, drawbacks to the use of toxic or thermal adjuvants. Phenol is considered cytotoxic with reported cases of hepatotoxicity and pulmonary edema and fibrosis. It is readily absorbable through the skin, mucosa, and open wounds. The acute lethal dose has been noted to be 1-2 g parenterally and 10 g dermally [17, 18]. Liquid Nitrogen has been associated with local tissue damage and risk of infection through particulate transfer [19]. Inhalational accidents have been reported with resultant acute serious upper airway injuries [20]. These growing concerns, as well as the financial burden of such practice, led to questioning the efficacy of these chemicals in reducing recurrence rates of GCT when used as local adjuvants. 

In a multicenter retrospective Scandinavian sarcoma study, 294 patients with GCT were followed for an average of 5 years [14]. The authors found cementation to be an effective method for reducing the recurrence rates after intralesional surgery. Similar results were reported by Becker et al. in their multicenter retrospective study of 298 patients treated between 1945 and 1998 [11]. Both studies showed a statistically significant difference favoring the use of PMMA as a local adjuvant. A recent study by Errani et al. found that “aggressive curettage” using a combination of phenol, alcohol and cement following intralesional surgery was associated with the lowest recurrence rate of the series of 12.5% [21]. 

In contrast, Blackley et al. reported on the recurrence rates of GCT in 59 patients between 1986 and 1996 treated with intralesional procedures using high-speed burr alone [7]. Mean follow up was 80 months and reported recurrence rate was 12%. They concluded that the adequacy of the removal of the tumor rather than the use of adjuvant modalities is what determines the risk of recurrence. In another multicenter study that included 186 patients, Turcotte et al. found no difference in the recurrence rates when comparing high speed burr alone to other local adjuvants [2]. Trieb et al. also found no significant difference in local recurrence rates with and without phenol as an adjuvant and emphasized the importance of adequate tumor removal [22].

Given the discrepant conclusions of the various studies on the surgical management of GCT with respect to the necessity of a chemical or thermal adjuvant to reduce local recurrence rates, the purpose of this study was to perform a systematic review of the literature and meta-analysis of the available data to compare the efficacy of toxic adjuvants and high-speed burring compared to high-speed burring alone in the surgical management of GCT.

## 2. Materials and Methods

### 2.1. Literature Search

A literature review was performed on all studies that assessed the effect of local adjuvant modalities on the recurrence rates of giant cell tumors. Searches through the Medline and EMBASE electronic databases were conducted through September 2009. The search was performed by two independent assessors and the results were compared. Keywords used in the search process included: giant cell tumor, bone, surgery, adjuvant, and recurrence. These were arranged using varying combinations of ‘‘AND,” ‘‘NOT,” and ‘‘OR,” and the results were limited to studies published or translated into the English language. Additional searches were performed manually through reference lists of review articles and relevant studies. Authors of potential eligible studies were also contacted to obtain unpublished data.

## 3. Inclusion and Exclusion Criteria

Studies were included if they reported on patients from the same cohort who were surgically treated for giant cell tumor of bone within two distinct comparison groups: one group treated with curettage and high speed burr followed by a local adjuvant, and the other group treated with curettage and high speed burr alone. Other inclusion criteria were studies that reported local recurrence as a primary outcome and those with at least 2 years of follow-up. Axial and appendicular tumor locations were included.

### 3.1. Quality Assessment

Eligible studies were evaluated by two independent reviewers for their quality using the Methodological Index for Nonrandomized Studies (MINORs) [23] scale and the Newcastle Ottawa Quality assessment scale (NOS) (http://www.ohri.ca/programs/clinical_epidemiology/oxford.htm). The scales allocate a maximum of nine points for quality of selection, comparability, exposure, and outcome of study participants.

### 3.2. Outcome Measures

The primary outcome targeted for analysis was the local recurrence rate, defined as radiological and pathological evidence of local disease recurrence necessitating further surgical intervention.

### 3.3. Statistical Analysis

The Comprehensive Meta-analysis version 1 software (Biostat Inc, NJ) was used for data analysis. Odds ratios (OR) and 95% confidence intervals (CI) are reported. Heterogeneity among studies was tested using the Cochrane Q test with a *P* *-*value set at a.1 for significance. The *I*-squared statistic is the percentage of total variation across studies due to heterogeneity. We had planned to use random effect model in the presence of heterogeneity and fixed effect model otherwise. A meta-analysis of pooled odds ratios was performed and an alpha of 0.05 was considered a criterion for statistical significance.

## 4. Results

### 4.1. Literature Search

**Figure 1:**
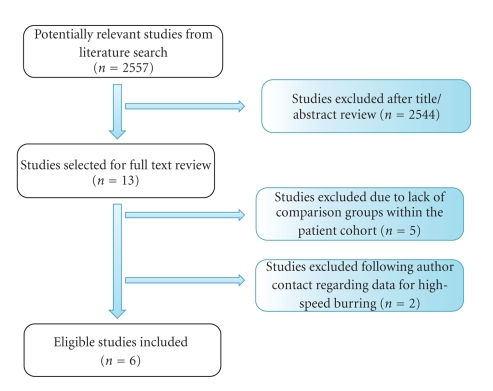
Literature search flow diagram.

Our initial search through the electronic databases yielded 2557 titles. After manual review of titles and abstracts, 2544 articles were excluded and 13 articles were determined relevant. Full text review was performed and five studies were excluded as they did not meet the inclusion criteria (appropriate comparison groups not present within the patient cohort). Two further studies were excluded following corresponding author contact due to unavailable original patient data regarding the use of a high-speed burr [11, 14]. Therefore, six studies were found to meet the criteria and were included in this meta-analysis (Figure 1) [2, 3, 9, 12, 22, 24].

### 4.2. Study Characteristics and Methodological Assessment

All six studies evaluated adult patients diagnosed with primary or recurrent giant cell tumor of bone treated with curettage and high speed burr with or without a local adjuvant. Adjuvant modalities used varied between phenol, PMMA, and liquid nitrogen (Table 1). Ninety-six percent of the patients included in the papers were treated for lesions of the appendicular skeleton. The level of agreement was very high between the two reviewers with median score of 7 for both reviewers using Newcastle-Ottawa questionnaire and median score of 7.5 for both reviewers using the MONIRs tool.

### 4.3. Local Recurrence

**Figure 2:**
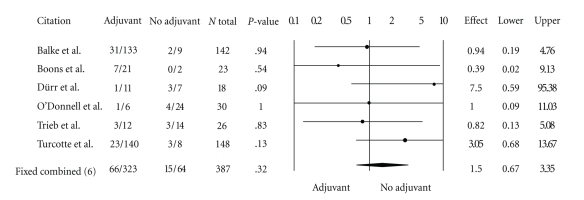
Forest plot for odds ratios of overall recurrence by type of intervention (95% CI, 0.67–3.35,  *P* = .32).

A total of 387 patients were included in the analysis, 323 treated with high speed burring and a local adjuvant and 64 patients treated with curettage and high speed burring alone. Sixty-six patients (20%) in the adjuvant group suffered a local recurrence while 15 patients (23%) in the no-adjuvant group suffered a local recurrence. The odds ratio in favor of no adjuvant therapy for local recurrence using both random and fixed effect models was 1.5 (95% CI, 0.67–3.35,  *P* = .32) (Figure 2).

### 4.4. Heterogeneity and Publication Bias

**Figure 3:**
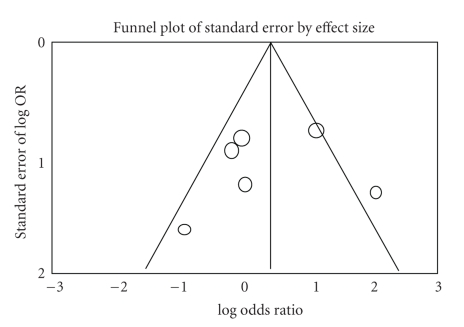
The funnel plot for the 6 studies included in the meta-analysis. The plot shows asymmetrical distribution indicating some publication bias in favor of studies showing an effect for the use of an adjuvant (to the left).

The variability (*I*-squared) in results across studies due to the true differences in treatment effect was 0%, which indicates no heterogeneity. The funnel plot for all studies is asymmetrical (Figure 3) indicating some publication bias in favor of studies showing an effect for the use of an adjuvant.

## 5. Discussion

Despite an abundance of published series on the surgical management and outcomes of GCT of bone, there remains a lack of consensus with respect to the need for a surgical adjuvant when intralesional curettage is performed. We have systematically reviewed the literature and identified six studies in which two groups from the same patient cohort were treated with intralesional curettage and high-speed burring with or without a chemical or thermal adjuvant such as phenol, liquid nitrogen, and PMMA. Pooled data from 387 patients did not indicate improved local control with the use of surgical adjuvants. 

These findings are significant in light of recent publications claiming that chemical or thermal adjuvants are required to reduce local recurrence in GCT of bone. Errani et al. reviewed a large series from a single institution in which the recurrence rate for the 200 patients who underwent intralesional curettage was 16% [21]. The authors defined “aggressive curettage” as one in which three local adjuvants were used (phenol, alcohol, and cement) and “standard curettage” as one in which phenol or alcohol was used but not cement (allograft or autograft was used to fill the defect). The recurrence rate for the “aggressive curettage” group was 12.5% and for the “standard curettage” group was 18%. These results were not statistically significant and the study did not have a comparable group in which no adjuvants were used. Kivioja et al. [14] and Becker et al. [11] published multicenter studies in 2008 strongly concluding that PMMA must be used to decrease recurrence rates in GCT of bone. However, neither study was able to identify the patients in the various treatment groups who underwent high-speed burring and those that did not (author communications). Both papers spanned several decades and the use of high-speed burring likely coincided with the introduction of chemical adjuvants. Therefore, despite these publications, there remains no data to support the need for chemical adjuvants if high-speed burring is employed.

In fact, results of other large series indicate that with meticulous surgical technique and high-speed burring, local control rates are comparable to other studies. Blackley et al. reported a 12% recurrence rate in 59 patients treated with intralesional curettage, high-speed burring, and bone grafting [7]. Similarly, Prosser et al. reported a 19% recurrence rate in 137 patients treated with curettage and high-speed burring alone [25]. Finally, the multicenter Canadian study by Turcotte et al. reported a recurrence rate of 18% for intralesional procedures in 148 patients and found that the nature of the filling material used or the use of an adjuvant failed to show any statistical impact on the recurrence risk [2]. Thus the conclusion that adjuvants such as cement are necessary to reduce recurrence in GCT of bone can be debated by the results of these cohort studies.

The ideal forum to answer the question as to whether an adjuvant is required would be a blinded randomized controlled trial. However, due to the logistics of performing such a study, the only available data at this time is observational as opposed to randomized. At the beginning of the millennium, the *New England Journal of Medicine* published two comprehensive studies comparing the point estimates and confidence intervals for various treatment outcomes published in the medical literature [26, 27]. The authors found convincing evidence that well-designed observational studies do not overestimate treatment effects and therefore pooled observational data can lead to valuable conclusions if randomized data is not available. The current meta-analysis showed some heterogeneity between studies (although without statistical significance) and all studies were evaluated as having acceptable methodology based on patient selection, comparability, and exposure. The reason for failing to detect a significant heterogeneity between studies is most likely due to the fact that the included patients, particularly in the “no-adjuvant” group, are small and the confidence intervals in the smaller studies are very wide. The pooling of the data, however, serves to narrow the confidence intervals and add more precision to the point estimates. In addition, the funnel plot indicates publication bias in favor of a surgical adjuvant, highlighting the importance of the nonsignificant effects of adjuvants in the pooled analysis.

In summary, we have systematically identified all studies in the literature reporting recurrence rates in GCT following intralesional curettage and high-speed burring with a comparison group from the same cohort who underwent the same procedure plus a chemical or thermal adjuvant. Despite possible publication bias in favor of adjuvants, our results support those of several groups who have concluded that meticulous surgical technique including high-speed burring is the most important step in reducing recurrence rates in GCT of bone. Surgical procedures will remain the preference of the individual surgeon. However, the claim that adjuvants such as phenol and/or PMMA are necessary to reduce recurrence rates is in fact not supported in the literature as demonstrated by this work.

## Figures and Tables

**Table 1:** Characteristics of included studies.

	Balke et al. [3]	Boons et al. [24]	Dürr et al. [12]	O'Donnell et al. [9]	Trieb et al. [22]	Turcotte et al. [2]
Year of publication	2008	2002	1999	1994	2001	2002
Follow up (months)	Mean 59.8	Mean 84	Median 61	Average 48	Median 132	Average 57
Ave Age (years)	33.4 years	34 years	33.5	31	33.5	36
Type of adjuvant	PMMA	PMMA + Liquid Nitrogen	Phenol	Phenol	Phenol	Phenol, PMMA and Liquid Nitrogen
% axial lesions	6.5%	8%	0%	0%	15%	0%

(PMMA: Polymethyl methacrylate).
